# Suppression of Fusarium Wilt in Watermelon by *Bacillus amyloliquefaciens* DHA55 through Extracellular Production of Antifungal Lipopeptides

**DOI:** 10.3390/jof9030336

**Published:** 2023-03-09

**Authors:** Dhabyan Mutar Kareem Al-Mutar, Noor Salih Abduljaleel Alzawar, Muhammad Noman, Azizullah, Dayong Li, Fengming Song

**Affiliations:** 1 Key Laboratory of Crop Diseases and Insect Pests of Ministry of Agriculture, Institute of Biotechnology, Zhejiang University, Hangzhou 310058, China; 2 Key Laboratory of Biology of Crop Pathogens and Insects of Zhejiang Province, Institute of Biotechnology, Zhejiang University, Hangzhou 310058, China; 3 Basra Agriculture Directorate, Almudaina 61008, Basra Province, Iraq; 4 College of Agriculture, Basra University, Albasra 61001, Basra Province, Iraq

**Keywords:** antifungal activity, biocontrol, *Fusarium oxysporum* f. sp. *niveum*, watermelon Fusarium wilt

## Abstract

Fusarium wilt caused by *Fusarium oxysporum* f. sp. *niveum* is one of the most devastating fungal diseases affecting watermelon (*Citrullus lanatus* L.). The present study aimed to identify potent antagonistic bacterial strains with substantial antifungal activity against *F. oxysporum* f. sp. *niveum* and to explore their potential for biocontrol of Fusarium wilt in watermelon. Out of 77 isolates from watermelon rhizosphere, six bacterial strains—namely, DHA4, DHA6, DHA10, DHA12, DHA41, and DHA55—exhibited significant antifungal activity against *F. oxysporum* f. sp. *niveum*, as well as other phytopathogenic fungi, including *Didymella bryoniae*, *Sclerotinia sclerotiorum*, *Fusarium graminearum*, and *Rhizoctonia solani*. These Gram-positive, rod-shaped, antagonistic bacterial strains were able to produce exo-enzymes (e.g., catalase, protease, and cellulase), siderophore, and indole-3-acetic acid and had the ability to solubilize phosphate. In greenhouse experiments, these antagonistic bacterial strains not only promoted plant growth but also suppressed Fusarium wilt in watermelon. Among these strains, DHA55 was the most effective, achieving the highest disease suppression of 74.9%. Strain DHA55 was identified as *Bacillus amyloliquefaciens* based on physiological, biochemical, and molecular characterization. *B. amyloliquefaciens* DHA55 produced various antifungal lipopeptides, including iturin, surfactin, and fengycin, that showed significant antifungal activities against *F. oxysporum* f. sp. *niveum*. Microscopic observations revealed that *B. amyloliquefaciens* DHA55 exhibited an inhibitory effect against *F. oxysporum* f. sp. *niveum* on the root surface of watermelon plants. These results demonstrate that *B. amyloliquefaciens* DHA55 can effectively promote plant growth and suppress the development of watermelon Fusarium wilt, providing a promising agent for the biocontrol of Fusarium wilt in watermelon.

## 1. Introduction

Watermelon (*Citrullus lanatus* L.) is an important tropical/subtropical horticultural crop that is cultivated worldwide due to its high nutritional and economic value. The greenhouse mono-cropping system, particularly in China, exacerbates the occurrence of destructive soil-borne diseases, including Fusarium wilt, anthracnose, and gummy stem blight [1,2]. Among these, Fusarium wilt, caused by the soil-borne fungus *Fusarium oxysporum* f. sp. *niveum* (*Fon*), has become a major constraint on the global watermelon industry [1]. Under severe infection conditions, Fusarium wilt can cause up to 100% yield losses in watermelon production [1]. Ineffective control measures and the long-term survival ability of the pathogen in soil make it impossible to manage Fusarium wilt. Therefore, research is urgently required to develop robust, sustainable, and eco-safe green strategies to manage and control destructive soil-borne diseases affecting watermelon, including Fusarium wilt.

Biological control of soil-borne diseases is well-established, and it is an environment-friendly alternative to traditional chemical methods [3]. Numerous studies have characterized a large number of microorganisms with the inherent potential for pathogen control [4,5,6,7,8,9,10,11]. For example, antagonistic microorganisms, such as *Penicillium oxalicum*, *Pseudomonas fluorescens*, *Streptomyces goshikiensis*, and *Bacillus amyloliquefaciens*, have been found to be effective in controlling/reducing Fusarium wilt development in watermelon [4,5,6,7,8,9,10,11]. Moreover, *Paenibacillus polymyxa* strains SQR-21 and WR-2 have been reported to exhibit plant growth-promoting activities, along with the production of antifungal metabolites that suppress Fusarium wilt in watermelon [4,5]. Similarly, *Bacillus subtilis* IBFCBF-4, *B. amyloliquefaciens* L3, and *Bacillus velezensis* F21 showed obvious antifungal activities against *Fon* and provided biocontrol efficiencies of 51% and 80%, respectively, against watermelon Fusarium wilt under greenhouse conditions [2,6,7]. However, studies on the biocontrol of watermelon Fusarium wilt that would make it possible to dissect the underlying disease-control mechanisms are still in progress.

Extensive research has been undertaken to discover the biocontrol mechanisms employed by antagonistic microbes against target pathogens. The antagonistic mechanisms operating against devastating fungal diseases include the production of antimicrobial compounds, modulation of the soil microbiome, and activation of induced systemic resistance response. Among these, the production of antifungal compounds is the primary mechanism of direct pathogen inhibition by which microbial antagonists inhibit phytopathogens and suppress plant diseases. *Bacillus* species produce a wide array of lipopeptides, such as bacillomycin D, iturin A, and fengycin, that function as antifungal compounds towards fungal pathogens [12,13,14,15]. For example, *B. amyloliquefaciens* strains VB7, MEP218, and ARP23 have been reported to produce different metabolic compounds, including iturinA, bacilysin, bacillomycin D, surfactin, subtilin, and subtilosin, that suppress stem rot disease in soybean and carnation caused by *Sclerotinia sclerotiorum* [16,17]. However, limited reports are available regarding the biocontrol mechanisms of potent microbes against Fusarium wilt. Given these facts, in-depth profiling of the antimicrobial metabolites produced by antagonistic microbes will open up new vistas for the development of robust and sustainable green strategies to control Fusarium wilt.

The present study aimed to identify and evaluate the antifungal efficacy of bacterial strains against *Fon* and investigate their potential as biocontrol agents against Fusarium wilt in watermelon. As a result of these efforts, six bacterial antagonists were obtained with efficacy against *Fon* and other pathogenic fungi, including *Didymella bryoniae* (*Db*), *Sclerotinia sclerotiorum* (*Ss*), *Fusarium graminearum* (*Fg*), and *Rhizoctonia solani* (*Rs*). These antagonistic bacterial strains were capable of promoting the growth of watermelon plants and reducing the disease severity of watermelon Fusarium wilt under greenhouse conditions. Among these antagonists, *B. amyloliquefaciens* DHA55 showed excellent potential for biocontrol of watermelon Fusarium wilt through its production of extracellular lipopeptides, and it can be used as an efficient biocontrol agent for the management of Fusarium wilt in watermelon.

## 2. Materials and Methods

### 2.1. Soil Sampling and Bacterial Isolation

Soil samples were collected from the rhizospheres of watermelon plants grown on different agricultural lands in Hangzhou, Zhejiang province, China, and stored at 4 °C until use. To isolate bacterial strains, 20 g of rhizosphere soil was suspended in 20 mL of sterile distilled water (ddH_2_O), vortexed vigorously, and placed in a water bath for 15 min at 100 °C. After heat treatment, the soil suspension was allowed to cool to room temperature and then serially diluted. An aliquot of 100 µL of the suspension (10^−4^ dilution) was spread on a Luria-Bertani (LB) agar plate and then incubated at 28 ± 2 °C for 36 h. Purified bacterial isolates were maintained on fresh LB plates.

### 2.2. Screening of Antagonistic Bacterial Strains

The tested pathogenic fungi included *Fusarium oxysporum* f. sp. *niveum* (*Fon*), *Didymella bryoniae* (*Db*), *Sclerotinia sclerotiorum* (*Ss*), *Fusarium graminearum* (*Fg*), and *Rhizoctonia solani* (*Rs*), which were obtained from the Laboratory of Crop Diseases and Insect Pests of the Ministry of Agriculture and Rural Affairs at Zhejiang University. The fungal pathogens were grown on potato dextrose agar (PDA) at 28 ± 2 °C for 5~6 d before the antagonistic experiments. The inhibition zone method was used to determine the antagonistic potential of the bacterial isolates against the indicated fungal pathogens as previously described [18]. Briefly, culture discs (5 mm in diameter) of each tested fungus were separately placed at the center of the PDA plates, and four discs (5 mm in diameter) of sterilized filter paper dipped in bacterial cultures with a concentration of 1 × 10^9^ colony-forming units (CFUs)/mL or in sterile LB medium (as controls) were placed at a distance of 2.5 cm from the fungal discs. After incubation at 28 ± 2 °C for 5~6 d, the inhibition zone was recorded and the mycelial growth inhibition rate was calculated using the following formula [18]:

$$\mathrm{Inhibition}\;\mathrm{rate}\;\left(\%\right)=\frac{\left(A\;-\;B\right)}{A}\times 100$$

where A is the diameter of the fungal colonies grown in the control, and B is the diameter of the fungal colonies grown with the bacterial culture. The experiments were conducted three times, and at least three replicates were included in each of the treatments.

### 2.3. Antifungal Activity of Cell-Free Filtrates of the Screened Antagonistic Bacterial Strains

The cell-free supernatant of the antagonistic bacterial strains was examined for its antifungal activity against *Fon* [19]. The bacterial strains were grown in 150 mL of LB media at 200 rpm and 28 ± 2 °C for 2 d as previously described [20], with minor modifications. After incubation, the bacterial culture supernatant was obtained through centrifugation at 20,000 rpm for 15 min at 4 °C followed by filtration through a 0.20 μm syringe filter. The PDA plates supplemented with 30 µL of filter-sterilized cell-free supernatant or the same volume of sterile LB medium (as controls) were inoculated with *Fon* mycelial discs (5 mm) and then incubated at 28 ± 2 °C for 5 d. The growth inhibition rate was calculated by comparing the diameters of the *Fon* colonies grown in the cell-free supernatant-containing plates to those grown in the control plates. The experiments were conducted three times, and at least three replicates were included in each of the treatments.

### 2.4. Physiological and Biochemical Characterization of the Antagonistic Bacterial Strains

The phosphate (P) solubilization activity of the antagonistic bacterial strains was determined using the Pikovskaya agar assay [21], and phosphate solubilization efficiency was calculated as described previously [22]. Indole-3-acetic acid (IAA) production was quantified according to a previously reported method [23]. Briefly, bacterial strains were cultured in LB medium amended with tryptophan (100 mg/L) under shaking conditions at 200 rpm and 28 ± 2 °C for 3 d, and 2 mL of the bacterial culture was centrifuged (10,000 rpm) at 4 °C. The bacterial pellet was resuspended in 4 mL of Salkowski reagent, and IAA production, as indicated by the development of a pink color, was determined using the colorimetric method as described previously [24]. Siderophore production was determined according to a previously described method [25]. The production of hydrogen cyanide (HCN) was measured as previously described [26]. Briefly, 100 µL in volume of bacterial culture was inoculated in LB medium supplemented with glycine (4.4 g/L). Sterile filter papers were saturated with two reagents (2% Na_2_CO_3_ and 0.5% C_6_H_3_N_3_O_7_) and fixed on the inner side of the plate covers. The plates were sealed with parafilm and incubated at 28 ± 2 °C for 4 d. Control plates were maintained without bacterial culture. For the ammonia production assay, the overnight bacterial culture was inoculated into 10 mL of LB broth and incubated at 30 ± 2 °C under shaking conditions (180 rpm) for 48 h. A total of 500 µL in volume of Nessler’s reagent was added to 10 mL of LB culture to measure the production of ammonia [27]. The activities of protease and catalase were determined according to methods described previously [28,29]. The experiments were conducted three times, and at least three replicates were included in each of the treatments.

### 2.5. Growth Promotion and Disease Suppression Assays

Watermelon (*C. lanatus* L. cv. Zaojia) seeds were imbibed in sterile ddH_2_O at 50 °C for 40 min and then incubated on moist cheesecloth in the dark at 28 ± 2 °C for 3 d for germination. The germinated seeds were transferred to pots (12 cm × 10 cm) filled with a sterilized mixture of perlite:vermiculite (1:2). After planting, watermelon plants were grown in a growth chamber at 70% humidity with a photoperiod of 16 h at 28 °C in the daytime and 20 °C at night. Watermelon plants were irrigated with 0.5× Hoagland solution (pH 6.5) once a week [30]. The bacterial strains were cultured in LB broth at 28 ± 2 °C and 180 rpm for 48 h and the inoculum suspension was prepared at a concentration of 1 × 10^9^ CFUs/mL. Two-week-old plants were used for growth experiments and separately supplied with 15 mL inoculum suspensions of the screened bacterial strains or with sterile LB medium as controls. At 15 d after treatment, plants were uprooted and washed gently with tap water to remove adhered soil particles, after which the growth parameters were estimated, including plant/root lengths and fresh/dry weights.

For disease assays, *Fon* was grown in mung bean broth medium (20 g/L mung bean boiled for 20 min, pH 7.0) at 28 ± 2 °C under shaking conditions at 150 rpm for 48 h. The spore suspension was adjusted to 4 × 10^6^ spores/mL for inoculation. Watermelon plants at the two-leaf stage were treated through soil drenching with 15 mL inoculum suspensions of each of the bacterial strains, pesticide Topsin-M (as a positive control), or sterile LB medium (as a negative control). At 5 d post-treatment, the bacterial strain-, Topsin-M- or LB-treated plants were inoculated with *Fon* by irrigating with a similar volume (15 mL) of spore suspension (4 × 10^6^ spores/mL) to assess their disease-controlling potential. The experiments were conducted three times using a completely randomized design and four replicates were included in each of the treatments. Disease scores were recorded using a five-scale rating standard (0 = healthy; 1 = ≤10% wilted; 2 = 11–20% wilted; 3 = 21–50% wilted; 4 = 50–100% wilted, and 5 = plants died). The disease severity index (DSI) was calculated as described previously [31], and biocontrol efficacy was determined using the following formula [32]:

$$\mathrm{Biocontrol}\;\mathrm{efficacy}\;=\frac{\left(\mathrm{DIA}\;-\;\mathrm{DIB}\right)}{\mathrm{DIA}}\times 100$$

where DIA represents the DSI for the control, and DIB is the DSI for the treatments.

### 2.6. DNA Extraction and Amplification of the 16S rRNA and Lipopeptide Biosynthesis Genes

Genomic DNA was extracted using a MiniBEST Bacteria Genomic DNA Extraction Kit Ver3.0 (Takara, Dalian, China) according to the manufacturer’s instructions. DNA concentration and quality were measured using a NanoDrop 2000 (Thermo-Fisher Scientific, Waltham, MA, USA). A fragment of the bacterial *16S rRNA* gene was amplified using a set of universal primers: 27F (5′-AGAGTTTGATCATGGCTCAG-3′) and 1479R (5′-TACGGTTACCTTGTTACGACTT-3′) [33]. For the amplification of the lipopeptide biosynthesis genes in the DHA55 strain, the *B. subtilis* strain SQR6 was used as a reference to design gene-specific primers (Table 1). The PCR products were separated on 1% (*w*/*v*) agarose gels and the target bands were purified using the AxyPrep DNA Gel Extraction Kit (AxyGene, Beijing, China) and then commercially sequenced (Zhejiang Youkang Biotech, Hangzhou, China). Homologues of the obtained sequences were searched for using the BlastN and BlastX programs at NCBI. The ClustalX program was used for multiple sequence alignment [34], and the phylogenetic tree was constructed using the neighbor-joining method with the MEGA 7.0 software package [35].

### 2.7. Extraction, Purification, and Antagonistic Activity of Lipopeptides

For the extraction of lipopeptides, strain DHA55 was grown in LB broth under shaking conditions at 200 rpm and 28 ± 2 °C for 3 d. The bacterial culture (100 mL) was centrifuged (12,000 rpm) at 4 °C for 20 min, and the lipopeptide-containing supernatant was collected. The lipopeptides were allowed to precipitate under acidic conditions (pH 2.0) using 2 M HCl and then incubated overnight at 4 °C. The precipitated lipopeptides were collected using centrifugation at 15,000 rpm at 4 °C for 20 min and then resuspended in CH_3_OH:H_2_O (2:1) solution. The extract was filtered through a 0.45 μm syringe filter, dried under a rotary vacuum at 40 °C, resuspended in 2 mL of methanol, and stored at −20 °C until further experiments. The antagonistic activity of the lipopeptide extract was determined as previously described [36]. Briefly, 60 µL of lipopeptide extract (i.e., ~30 µg/µL) was poured into the right and left wells (5 mm in diameter) of the PDA plate, and similar volumes of dimethyl sulfoxide (DMSO) were added in the remaining wells as controls. Mycelial discs of *Fon* (5 mm in diameter) were placed at the center of the PDA plates and incubated at 28 ± 2 °C for 3~5 d. The colony sizes were recorded, and the inhibition rate was determined by comparing the diameters of the fungal colonies grown with the lipopeptide extract to those grown with controls [37].

### 2.8. Minimal Inhibitory Concentration Assay

*Fon* was grown under agitating conditions in mung bean liquid medium at 28 ± 2 °C for 48 h, and spores were collected. Wells in 96-well microtiter plates were first filled with 50 µL of spore suspensions (4 × 10^5^ spores/mL) and then with lipopeptide extract to final concentrations of 50, 75, 100, 200, and 300 µg/mL or with DMSO (0 µg/mL) for the controls, following a previous report [38]. Fungal growth was estimated by measuring the OD_600_ values, and the growth inhibition rate was calculated by comparing fungal growth in lipopeptide suspensions to that in controls. The experiments were conducted three times, and at least three replicates were included in each of the treatments.

### 2.9. Matrix-Assisted Laser Desorption/Ionization–Time of Flight (MALD-TOF) Analysis

Lipopeptide extract was dissolved in 2 mL of methanol and subjected to MALD-TOF/mass spectrometry (MS) analysis to identify possible lipopeptides [39]. Briefly, 1 µL of lipopeptide extract was dripped onto the MALDI-TOF-MS target in a Bruker Daltonik Reflex. The Ultraflextreme instrument (Bruker Daltonics, Bremen, Germany) was used for desorption and ionization, with a 337 nm nitrogen laser directed at an Eppendorf tube containing the matrix solution (10 mg/mL in 70% of C_10_H_7_NO_3_, 30% of C_2_H_3_N, and 0.1% trifluroacetic acid). Spectral data were analyzed to identify the presence of different lipopeptides in the extract.

### 2.10. Gas Chromatography–Mass Spectrometry (GC-MS) Analysis of Fatty Acids

The strain DHA55 was cultured on Tryptic Soy Blood Agar (TSBA) medium (Difco, Detroit, MI, USA) at 28 ± 2 °C for 24 h. Lipids were extracted using the Sherlock Microbial Identification System. Lipid profiling was performed using a GC (Hewlett Packard HP 5890 Serie, Ramsey, MN, USA). The fatty acid methyl esters were identified based on their relative retention times compared to the standard of the methyl ester, 3-hydroxy-hexadecanoate (Sigma-Aldrich, St. Louis, MO, USA), and the fragmentation profile obtained using MS with electronic ionization (EI-70 eV). The fatty acids were determined by comparing their retention times to the whole area of the peak. The fatty acids of the bacterial strain DHA55 were identified by searching the Sherlock bacterial fatty acid reference library RTSBA6 6.10 [40].

### 2.11. Scanning Electron Microscope (SEM) Observation

The interaction between the bacterial strain DHA55 and *Fon* on cellophane membrane and on the root surface of watermelon plants was examined using an SEM (Model TM-1000, Hitachi, Tokyo, Japan). To observe the direct interaction with *Fon*, 5 µL in volume of *Fon* spore suspension (4 × 10^6^ spores/mL) was dropped onto the cellophane membrane placed on the PDA plate and incubated at 28 ± 2 °C for 72 h. Then, 30 µL in volume of bacterial suspension (~10^9^ CFUs/mL) was then dropped onto the *Fon* mycelia grown on the cellophane membrane and incubated at 28 ± 2 °C for 24 h. After incubation, samples were collected, washed with sterile ddH_2_O twice, and fixed with 2.5% glutaraldehyde. To examine the interactions presented by DHA55 on the watermelon root surface, plants were harvested carefully at the first true-leaf stage, washed with sterile ddH_2_O twice, soaked in 98% ethanol for 30 s, rinsed again with sterile ddH_2_O, and dried on sterile paper. The roots of the seedlings were then soaked in the suspension of the bacterial strain DHA55 (1 × 10^9^ CFUs/mL) and incubated at 28 ± 2 °C at 150 rpm for 2 h. The DHA55 strain-treated and untreated seedlings were then grown hydroponically in a *Fon* spore suspension (4 × 10^6^ spores/mL) under shaking conditions (95 rpm) at 28 ± 2 °C. *Fon*-inoculated and non-inoculated root samples with and without treatment with DHA55 strain were collected after 24 and 48 h, rinsed with sterile ddH_2_O, cut into 0.5–1.0 cm pieces, and fixed with 2.5% glutaraldehyde. All samples were prepared according to a previously described procedure for SEM analysis [41].

### 2.12. Statistical Analysis

Data analysis was conducted using one-way analysis of variance with the SPSS 20.0 software package. The Duncan multiple range test (*p*-value < 0.05) was performed to determine differences between the treatment means of the different datasets.

## 3. Results

### 3.1. Isolation of Antagonistic Bacterial Strains and Evaluation of Their Antifungal Activity against Pathogenic Fungi

**Figure 1:**
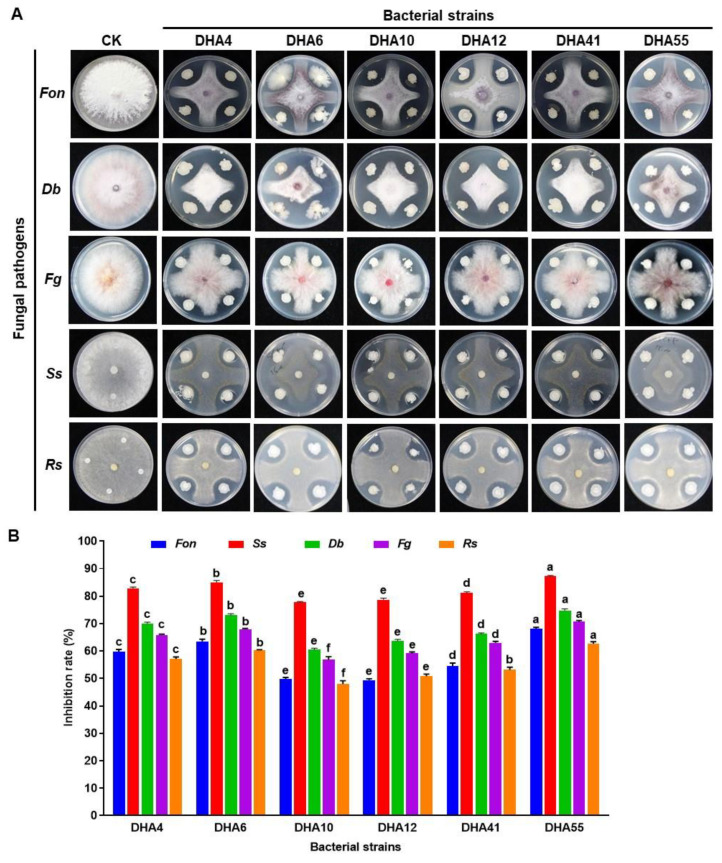
Antagonistic activity of bacterial strains against different pathogenic fungi. (**A**) Inhibition zones of bacterial strains against *Fusarium oxysporum* f. sp. *nevium* (*Fon*), *Didymella bryoniae* (*Db*), *Sclerotinia sclerotiorum* (*Ss*), *Fusarium graminearum* (*Fg*), and *Rhizoctonia solani* (*Rs*). (**B**) The inhibition rates (%) of bacterial strains against the pathogenic fungi. The results in (**A**) are from an experiment that was performed independently three times with similar results. The data presented in (**B**) are the means ± standard deviation from three independent experiments. Different letters above the columns indicate significant differences between the different bacterial strains for the same tested fungus at the *p*-value < 0.05 level according to the one-way analysis of variance test.

A total of 77 bacterial strains were isolated from the rhizospheres of field-grown watermelon plants. Six strains—namely, DHA4, DHA6, DHA10, DHA12, DHA41, and DHA55—showed significant inhibitory activity against five fungal pathogens, including *Fon*, *Ss*, *Db*, *Fg*, and *Rs* (Figure 1A). These six bacterial strains exhibited a variable inhibitory spectrum against the tested phytopathogenic fungi (Figure 1B). Among these strains, DHA55 was found to be the most proficient antagonistic strain, showing the maximum in vitro inhibition rate of 68% against *Fon* (Figure 1B). Strain DHA55 also exhibited the highest growth inhibition rates of 87.2%, 74.8%, 70.6%, and 62.7% against *Ss*, *Db*, *Fg*, and *Rs*, respectively (Figure 1A,B). Taken together, all antagonistic strains demonstrated inherent inhibition potential against phytopathogenic fungi; however, strain DHA55 outperformed other isolates owing to its better pathogen-inhibiting ability, especially against *Fon*.

**Figure 2:**
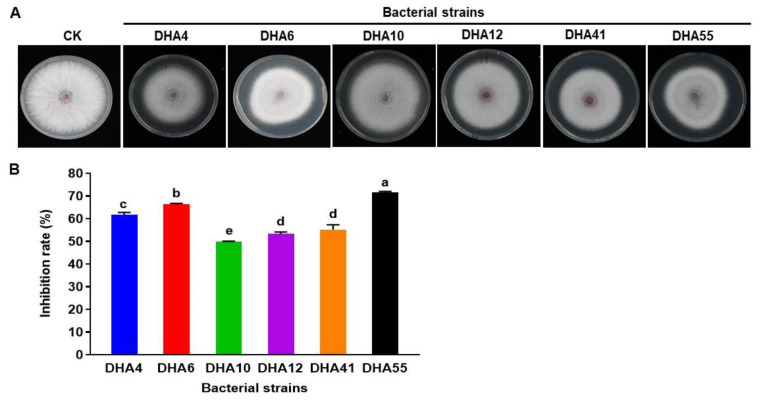
The antifungal activity of the cell-free filtrate of the antagonistic bacterial strains against *Fusarium oxysporum* f. sp. *nevium* (*Fon*). (**A**) *Fon* colonies were grown on potato dextrose agar supplemented with the cell-free filtrate of the antagonistic bacterial strains or with sterile Luria-Bertani medium as controls. (**B**) The inhibition rate (%) for the cell-free filtrate of the bacterial antagonists against *Fon*. The results in (**A**) are from an experiment that was performed independently three times with similar results. The data presented in (**B**) are the means ± standard deviation from three independent experiments. Different letters above the columns indicate significant differences at the *p*-value < 0.05 level according to the one-way the analysis of variance test.

The antifungal activity of the cell-free filtrate of the antagonistic bacterial strains was evaluated further against *Fon*. The results showed that the cell-free filtrate from these six bacterial strains inhibited the mycelial growth of *Fon* colonies (Figure 2A). Cell-free filtrate from bacterial strains DHA55 and DHA6 achieved the highest growth inhibition percentages of 71.5% and 66.2%, respectively, as compared to the control (Figure 2B). These results indicate that cell-free filtrates of the bacterial strains, especially those of DHA55 and DHA6, contain potent antimicrobial compounds that inhibit the radial growth of *Fon*.

### 3.2. Physiological and Biochemical Characterization of the Antagonistic Bacterial Strains

**Figure 3:**
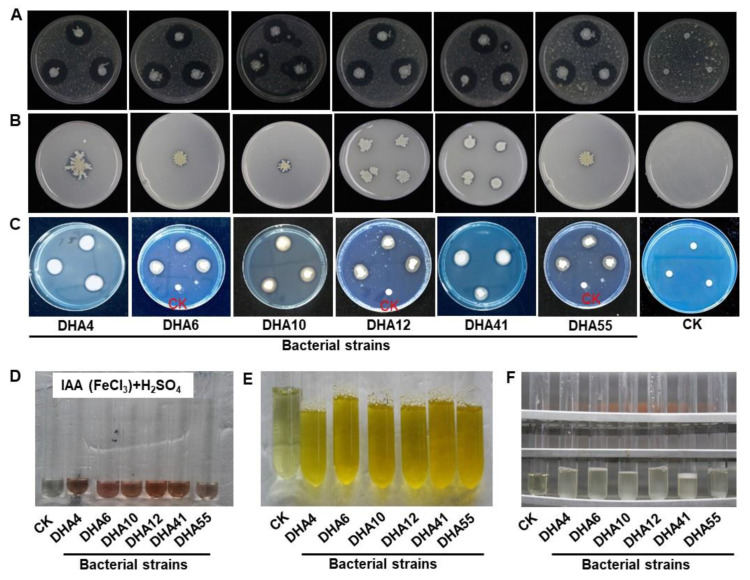
Biochemical characterization of the antagonistic bacterial strains. (**A**) Protein and glucan hydrolysis. (**B**) Phosphate solubilization. (**C**) Siderophore production. (**D**) Indole acetic acid (IAA) production. (**E**) Ammonium production. (**F**) Catalase activity. The controls (CK) were inoculated with sterile Luria-Bertani medium. The experiments were performed independently three times with similar results.

A series of biochemical and physiological tests were performed to characterize the antagonistic bacterial strains. The result showed that all antagonistic bacterial strains were rod-shaped and Gram-positive and produced catalase, protease, cellulase, and ammonium but not HCN (Table 2, Figure 3A,E,F). Furthermore, these antagonistic bacterial strains also produced IAA and siderophore and exhibited P solubilization activity (Table 2, Figure 3B–D). Among them, strain DHA55 produced 393.14 µg/mL of IAA (Table 2, Figure 3D) and showed a P solubilization zone of 0.40 ± 0.01 mm (Table 2, Figure 3B) and the highest degradation zone of 0.54 ± 0.20 mm for siderophore production (Table 2, Figure 3C). Overall, these results confirmed that the obtained antagonistic bacterial strains possessed a variety of plant growth-promoting biochemical and physiological features.

### 3.3. Growth-Promoting Activity of the Antagonistic Bacterial Strains

**Figure 4:**
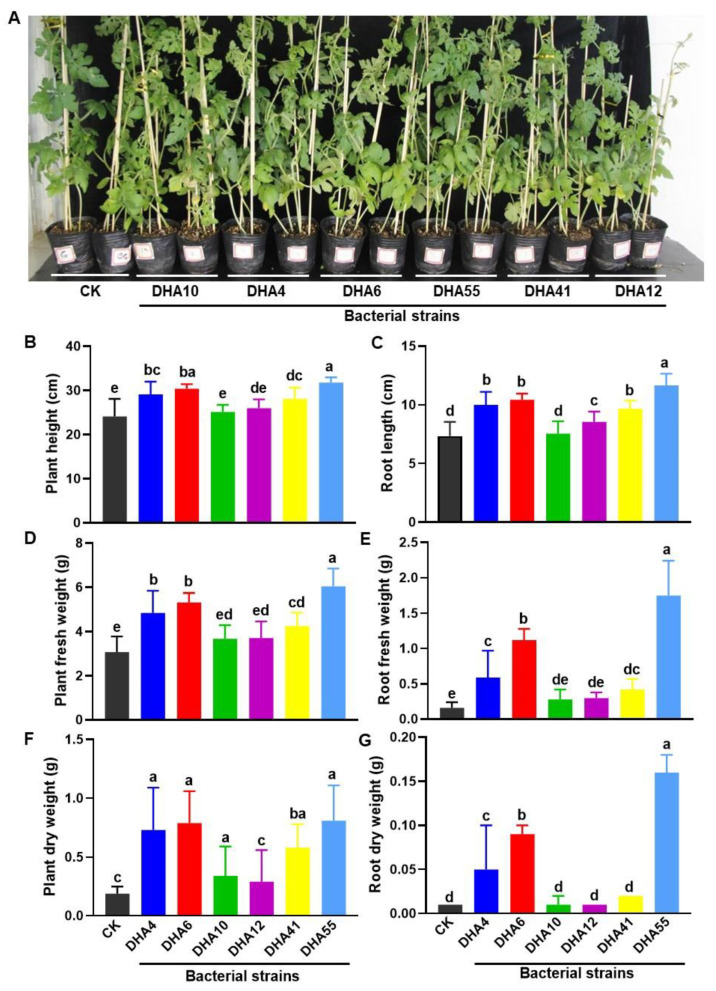
Growth-promoting activity of the antagonistic bacterial strains in watermelon plants. Watermelon plants were treated using root drenching with either sterile Luria-Bertani medium (CK) or 15 mL suspensions of each of the antagonistic bacterial strains. Photos and growth parameters were obtained at 30 d after treatment. (**A**) Growth performance of the watermelon plants with and without treatment with the antagonistic bacterial strains. (**B**,**C**) Height of aboveground plant and root length with and without treatment with the antagonistic bacterial strains. (**D**,**E**) Fresh weight of aboveground plants and roots with and without treatment with the antagonistic bacterial strains. (**F**,**G**) Dry weight of aboveground plants and roots with and without treatment with the antagonistic bacterial strains. The results in (**A**) are from an experiment that was performed independently three times with similar results. The data presented in (**B**–**G**) are the means ± standard deviation from three independent experiments. Different lowercase letters indicate a significant difference at *p*-value < 0.05 level according to the one-way analysis of variance test.

It is a well-known fact that most of the antagonistic bacteria isolated from plant rhizospheres have plant growth-promoting activity [42]. To examine whether the obtained antagonistic bacterial strains had growth-promoting abilities, the bacterial strains were individually applied to the experimental plants and the growth parameters of the inoculated watermelon plants were compared. Overall, the antagonistic bacterial strains showed growth-promoting abilities, as judged by the growth performance of the watermelon plants treated with each of the antagonistic bacterial strains (Figure 4A). The heights of the aboveground parts and the lengths of the roots in the inoculated watermelon plants ranged from 25.1 ± 1.36 cm~31.77 ± 1.2 cm and 8.6 ± 0.8 cm~11.66 ± 0.7 cm, respectively, which were significantly higher than those in the untreated watermelon plants (24.1 ± 3.9 cm plant height and 7.3 ± 1.2 cm root length) (Figure 4B,C). Similarly, the fresh and dry weights of the aboveground parts and roots in the inoculated watermelon plants were also significantly higher than those in the untreated watermelon plants (Figure 4D–G). Among the antagonistic strains, DHA55 was found to be most effective, and the overall growth-promoting activity of the antagonistic bacterial strains was found to be in the order DHA55 > DHA10 > DHA12 > DHA41 > DHA4 > DHA6 (Figure 4). These results suggested that the antagonistic bacterial strains, especially the DHA55 strain, could improve the growth performance of watermelon plants by increasing biomass production.

### 3.4. Biocontrol Efficacies of the Antagonistic Bacterial Strains against Watermelon Fusarium Wilt

**Figure 5:**
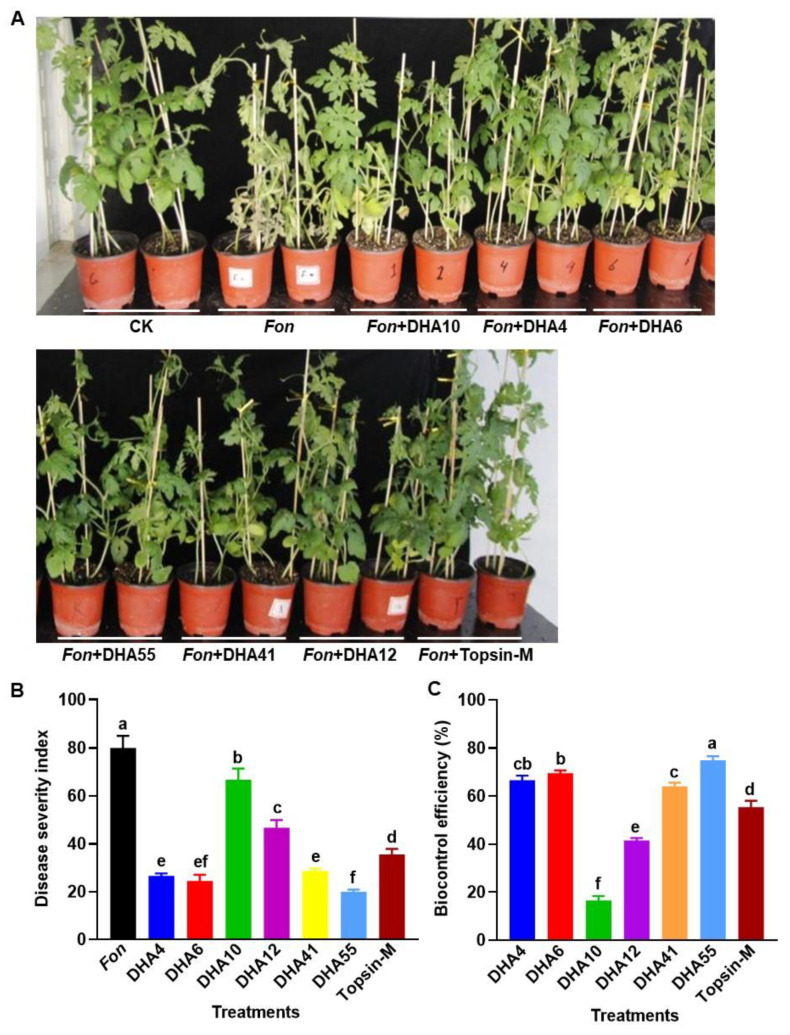
Suppression of watermelon Fusarium wilt by the antagonistic bacterial strains. (**A**) Disease phenotype of *Fusarium oxysporum* f. sp. *nevium* (*Fon*)-inoculated watermelon plants pretreated with the antagonistic bacterial strains, Topsin-M, or Luria-Bertani medium (CK) at 15 d post-inoculation. (**B**) Disease severity index of *Fon*-inoculated watermelon plants with or without the antagonistic bacterial strains. (**C**) Biocontrol efficacies of the antagonistic bacterial strains against Fusarium wilt in watermelon. Watermelon plants were treated by root drenching with 15 mL suspension of each of the antagonistic bacterial strains, Topsin-M (a positive control), or Luria-Bertani medium (CK), followed by *Fon* inoculation at 5 d after applying treatments. The results in (**A**) are from an experiment that was performed independently three times with similar results. The data presented in (**B**,**C**) are the means ± standard deviation from three independent experiments. Different lowercase letters indicate significant differences at the *p*-value < 0.05 level according to the one-way analysis of variance test.

To evaluate the biocontrol potential of the antagonistic bacterial strains against Fusarium wilt in watermelon, a series of disease assays were performed. At 15 d post-inoculation with *Fon*, typical disease symptoms, such as yellowing of vines and wilted leaves, began to appear in the watermelon plants pretreated with and without each of the antagonistic bacterial strains and Topsin-M, while no disease symptoms were observed in healthy controls (Figure 5A). Overall, the untreated *Fon*-inoculated plants showed more severe disease symptoms than the infected plants pretreated with the antagonistic bacterial strains or Topsin-M (Figure 5A). The DSI in the untreated *Fon*-inoculated plants reached ~80 at 35 d after inoculation, while the *Fon*-inoculated plants pretreated with the fungicide Topsin-M showed a reduced DSI of 35.55 ± 2.3 with a control efficiency of 55.36 ± 2.6% against Fusarium wilt (Figure 5B,C). The DSIs in the *Fon*-inoculated plants pretreated with of each of the antagonistic bacterial strains ranged from 19.9 ± 0.88 to 66.8 ± 4.7, which were significantly lower than those for the untreated *Fon*-inoculated plants (Figure 5B). Among the six antagonistic bacterial strains, the *Fon*-inoculated plants treated with the strain DHA10 showed the highest DSIs and the lowest biocontrol efficacy of 16.5 ± 1.8%, while the *Fon*-inoculated plants pretreated with strain DHA55 showed the lowest DSI and the highest biocontrol efficacy of 74.9% (Figure 5C). Overall, the biocontrol efficacies of the antagonistic bacterial strains against watermelon Fusarium wilt were in the order DHA55 > DHA6 > DHA4 > DHA41 > DHA12 > DHA10 (Figure 5C). These results indicated that the antagonistic bacterial strains had variable biocontrol potential against watermelon Fusarium wilt, with the DHA55 strain showing the maximum biocontrol efficiency; thus, it can be used as an efficient biocontrol agent to suppress Fusarium wilt in watermelon. Based on the preliminary results from the in vitro antifungal and disease assays, DHA55 was selected for further experiments.

### 3.5. Molecular Characterization of the Antagonistic Bacterial Strain DHA55

**Figure 6:**
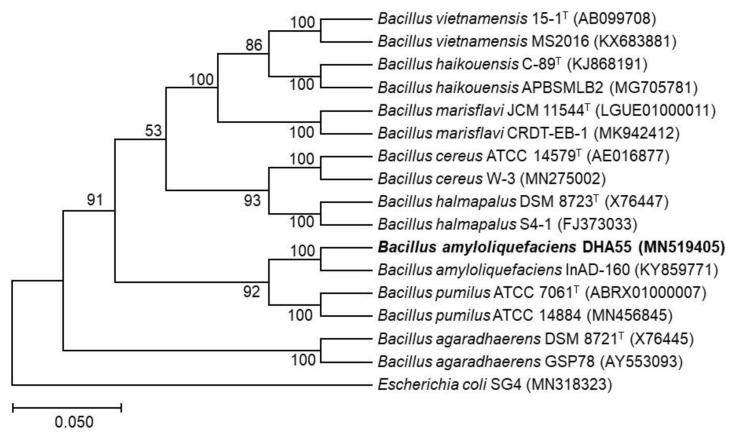
The phylogenetic tree of the *16S rRNA* gene sequence of strain DHA55 with those from other *Bacillus* species. MEGA7.0 software was used to construct the phylogenetic tree using the neighbor-joining method. *Escherichia coli* SG4 (MN318323) was used as an outgroup.

To taxonomically characterize the bacterial strain DHA55, the *16S rRNA* gene was amplified and sequenced. The phylogenetic analysis revealed that the *16S rRNA* gene sequence of the strain DHA55 (MN519405) was closely clustered with that of *B. amyloliquefaciens* InAD-160 (KY859771), showing 100% sequence identity (Figure 6). Therefore, it is likely that the antagonistic bacterial strain DHA55 belongs to the genus *Bacillus* and is a strain of *B. amyloliquefaciens*.

### 3.6. Antifungal Activity of Lipopeptides Produced by B. amyloliquefaciens DHA55

**Figure 7:**
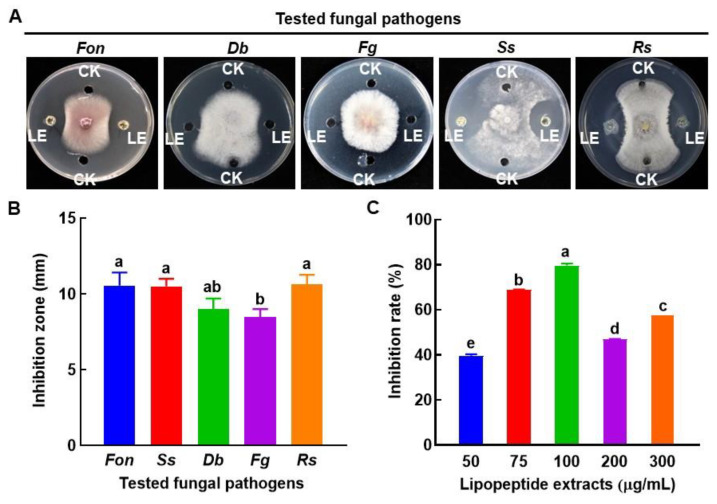
Inhibition activity of the lipopeptide extract of *Bacillus amyloliquefaciens* DHA55 against the mycelial growth of different pathogenic fungi. A total of 60 µL in volume of the lipopeptide extract (LE) was added into the right and left wells in potato dextrose agar plates, while 60 μL of dimethyl sulfoxide (CK) was added to the upper and lower control wells. Culture discs (5 mm in diameter) of the tested fungi were placed at the center of the plate. (**A**) Growth of the tested fungi. *Fon*, *Fusarium oxysporum* f. sp. *nevium*; *Db*, *Didymella bryoniae*; *Fg*, *Fusarium graminearum*; *Ss*, *Sclerotinia sclerotiorum*; *Rs*, *Rhizoctonia solani*. (**B**) The inhibition zone (mm) of the lipopeptide extract against the tested fungi. (**C**) The inhibition activity for varying levels of lipopeptide extract against *Fon* growth. The results in (**A**) are from an experiment that was performed independently three times with similar results. The data presented in (**B**,**C**) are the means ± standard deviation from three independent experiments. Different lowercase letters in the columns indicate significant differences at the *p*-value < 0.05 level according to the one-way analysis of variance test.

To investigate the possible biochemical mechanism behind the antifungal activity of *B. amyloliquefaciens* DHA55, lipopeptides were extracted and examined for their inhibitory activity against *Fon* and other fungal pathogens. The crude lipopeptide extract from *B. amyloliquefaciens* DHA55 exhibited significant inhibitory activities against the mycelial growth of *Fon*, *Ss*, and *Rs* (Figure 7A), with average inhibition zones of 10.5 mm, 10.5 mm, and 10.6 mm, respectively (Figure 7B). However, the lipopeptide extract did not significantly inhibit the mycelial growth of *Db* and *Fg* (Figure 7A,B).

The lipopeptide extract from *B. amyloliquefaciens* DHA55 inhibited *Fon* growth (Figure 7C). The highest *Fon* growth inhibition (79.4%) was observed at a concentration of 100 µg/mL of the lipopeptide extract, while the lipopeptide extracts at concentrations of 50 and 75 µg/mL also inhibited *Fon* growth by 39.4~68.7% (Figure 7C). Surprisingly, the growth inhibition effects with higher concentrations (200 and 300 µg/mL) of the lipopeptide extract against *Fon* were reduced compared to that at 100 µg/mL (Figure 7C). Together, these results indicated that the extracellular lipopeptides of *B. amyloliquefaciens* DHA55 had the ability to inhibit the mycelial growth of *Fon*.

### 3.7. Identification of the Lipopeptide Biosynthesis Genes in B. amyloliquefaciens DHA55

The biosynthesis of lipopeptides in *B. amyloliquefaciens* DHA55 was further confirmed using genetic amplification of the genes responsible for the biosynthesis of different lipopeptides, such as iturin, fengycin, and surfactin. As shown in Table 3, fragments with expected sizes (Figure S1) corresponding to three iturin genes (*Iturin B*, *C*, and *D*), two fengycin genes (*Fengycin E* and *D*), and one surfactin gene (*Surfactin*) were amplified from *B. amyloliquefaciens* DHA55. BLASTn searching revealed that the sequences of *Iturin C*, *Iturin D*, *Fengycin E*, and *Fengycin D* amplified from *B. amyloliquefaciens* DHA55 showed 98.60%, 95.81%, 97.31%, and 100% nucleotide identities with their corresponding genes in other *Bacillus* species, with *e* values of 0, 0, 0, and 8 × 10^−79^, respectively (Table 3). These results further confirmed the inherent genetic basis involved in the biosynthesis of lipopeptides in *B. amyloliquefaciens* DHA55.

### 3.8. Characterization of Lipopeptides Produced by B. amyloliquefaciens DHA55

**Figure 8:**
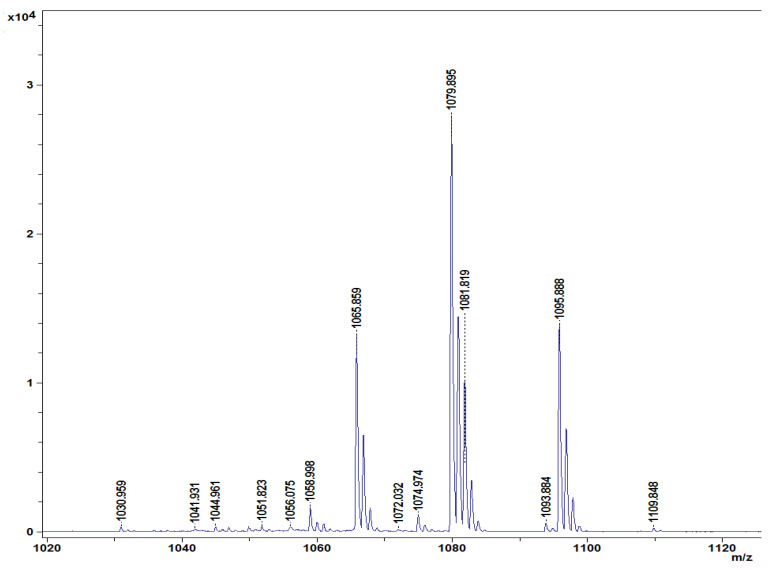
Characterization of lipopeptides produced by *Bacillus amyloliquefaciens* DHA55. Matrix-assisted laser desorption/ionization–time of flight profiles of lipopeptides extracted from *B. amyloliquefaciens* DHA55. Four families of lipopeptides corresponding to different characteristic peaks marked in *m*/*z*—fengycin (*m*/*z*: 1030.959, 1058.998, and 1074.974), iturin (*m*/*z*: 1079.895 and 1093.884), surfactin (*m*/*z*: 1044.961 and 1065.859), and bacillomycin D (iturin family; *m*/*z*: 1095.522)—were identified.

Different lipopeptides with peaks in the mass spectrum from 1030 to 1109 *m*/*z* were identified in the lipopeptide extract of *B. amyloliquefaciens* DHA55 using MALDI-TOF analysis (Figure 8). The highest spectral peak for *B. amyloliquefaciens* DHA55 was typically associated with non-ribosomal lipopeptides. Four families of lipopeptides, including surfactin (with peaks at 1044.961 and 1065.859 *m*/*z*), iturin (with peaks at 1079.895 and 1093.884 *m*/*z*), bacillomycin D (iturin family; with a peak at 1095.522 *m*/*z*), and fengycin (with peaks at 1030.959, 1058.998, and 1074.974 *m*/*z*), were detected (Figure 8). The contents of the different cellular fatty acids were also measured. C15:0 anteso, C15:0 iso, C16:0 iso, and C16:0 were found to be the major branched fatty acids in *B. amyloliquefaciens* DHA55, while C14:0 iso, C17:0 anteiso, and C17:0 iso were found to be the minor branched fatty acids (Table 4). These results indicated that *B. amyloliquefaciens* DHA55 was capable of producing a range of lipopeptides bearing antifungal activity against different phytopathogenic fungi; in particular, against *Fon*.

### 3.9. Colonization of B. amyloliquefaciens DHA55 on Watermelon Roots

**Figure 9:**
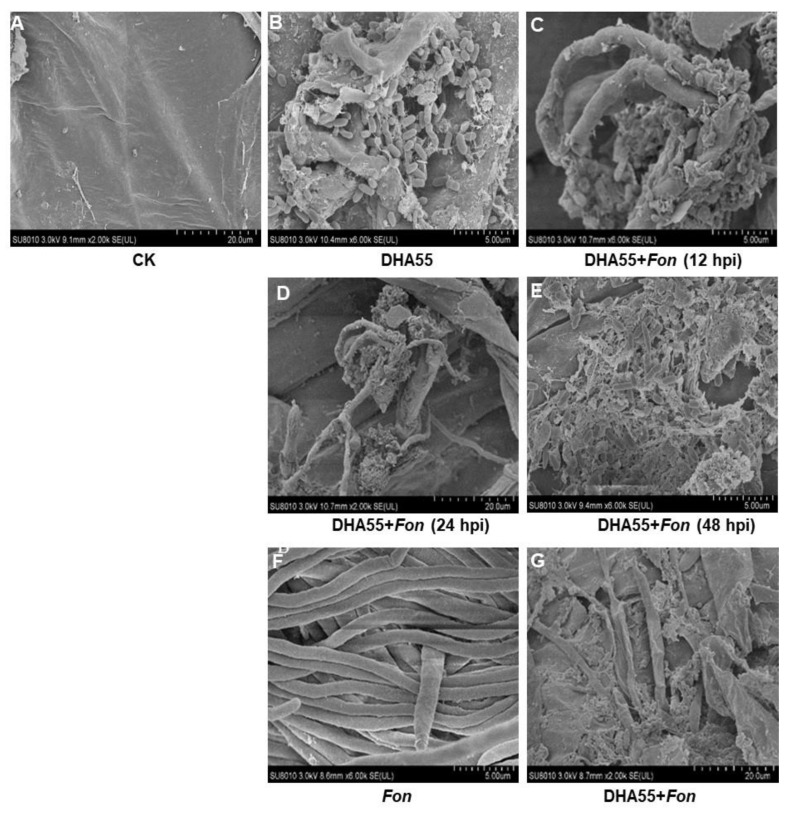
Direct interaction between *Bacillus amyloliquefaciens* DHA55 and *Fusarium oxysporum* f. sp. *nevium* (*Fon*) on watermelon roots and cellophane membrane, as revealed by SEM observations. (**A**) Root surface of watermelon plants treated with sterile Luria-Bertani medium (CK). (**B**) Colonization of *B. amyloliquefaciens* DHA55 on watermelon root surface at 24 h after inoculation. (**C**) Adherence of *B. amyloliquefaciens* DHA55 to *Fon* mycelia on the root surface of watermelon plants at 12 h after inoculation. (**D**,**E**) *B. amyloliquefaciens* DHA55 disrupted *Fon* mycelia on the root surface of watermelon plants at (**D**) 24 and (**E**) 48 h after inoculation, respectively. (**F**) *Fon* mycelia grown on cellophane membrane without *B. amyloliquefaciens* DHA55 treatment. (**G**) *B. amyloliquefaciens* DHA55 caused damage to *Fon* mycelia grown on cellophane membrane. The watermelon plants were grown hydroponically, and roots were washed gently with water before imaging. The experiments were independently performed three times with similar results.

Colonization of *B. amyloliquefaciens* DHA55 on the watermelon root surface and its interaction with *Fon* were studied by SEM. DHA55-uninoculated watermelon plants showed a smooth root surface without bacterial colonization (Figure 9A), while *B. amyloliquefaciens* DHA55 efficiently colonized and proliferated on the root surface of the DHA55-inoculated plants (Figure 9B). When co-inoculated, *B. amyloliquefaciens* DHA55 adhered to the *Fon* mycelia on the root surface of watermelon plants at 12 h after inoculation, and the *Fon* mycelia were destroyed and eventually collapsed at 48 h after inoculation (Figure 9C–E). The contact mortality of *Fon* in the presence of *B. amyloliquefaciens* DHA55 was further confirmed in an in vitro assay. In the absence of *B. amyloliquefaciens* DHA55, *Fon* grew well with normal mycelia on the cellophane membrane on PDA (Figure 9F); however, in the presence of *B. amyloliquefaciens* DHA55, the *Fon* mycelia were destroyed and collapsed (Figure 9G). These observations indicated that *B. amyloliquefaciens* DHA55 could successfully colonize the root surface of watermelon plants and antagonize *Fon* colonization, reducing disease incidence.

## 4. Discussion

Rhizosphere-inhabiting *Bacillus* species have been shown to promote plant growth and suppress disease occurrence in a variety of crop plants, providing eco-friendly and cost-effective alternatives for crop disease management. This study identified six antagonistic bacterial strains that showed significant antifungal activity against different fungal pathogens (Figure 1) and displayed great potential for plant growth promotion and Fusarium wilt suppression in watermelon (Figure 3, Figure 4 and Figure 5). Specifically, *B. amyloliquefaciens* DHA55 exhibited the best biocontrol efficacy against Fusarium wilt in watermelon and produced diverse antifungal lipopeptides against *Fon*. These results provide a basis for the development of *B. amyloliquefaciens* DHA55-based formulations for the biocontrol of watermelon Fusarium wilt under field conditions.

Biocontrol agents possess distinct phytobeneficial biochemical and physiological mechanisms, such as the production of phytohormones, siderophores, extracellular enzymes, and antimicrobial compounds, that allow them to regulate plant growth and stress tolerance under adverse environmental conditions [43,44,45,46,47,48,49]. In the present study, the antagonistic bacterial strains secreted a number of extracellular enzymes, including catalase, protease, and cellulase (Table 2, Figure 3), that might be involved in the lysis of fungal mycelia [50,51]. Conversely, the capability of the antagonistic bacterial strains, especially *B. amyloliquefaciens* DHA55, to produce siderophores, ammonium, and IAA and to solubilize inorganic P (Table 2, Figure 3) might be linked to their growth-promoting activity in watermelon plants (Figure 4). This is in line with previous reports that *Bacillus* species, including some *B. amyloliquefaciens* strains, enhanced the growth of rice and wheat plants and displayed plant growth-promoting traits; e.g., P solubilization and IAA, siderophore, and ammonium production [52,53,54,55]. Considering these results, the unique and superior phytobeneficial traits of *B. amyloliquefaciens* DHA55 can be exploited under actual field conditions to improve plant growth.

Based on biochemical, physiological, and molecular characterization (Table 2, Figure 6), the most effective phytobeneficial bacterial strain, DHA55, was identified as *B. amyloliquefaciens*, which was further confirmed by the detection of relatively high levels of the characteristic fatty acids 15:0iso and 15:0anteso (Table 4). Previous studies have shown that *B. amyloliquefaciens* strains possess significant antifungal activity against *F. oxysporum*, which suppresses Fusarium wilt in various crops, such as watermelon and tomato [50,52,53,54,55,56,57]. Moreover, the cell-free supernatant of the *B. amyloliquefaciens* DHA55 culture displayed significant growth-inhibiting potential against different pathogenic fungi, including *Fon*, *Db*, *Fg*, *Ss*, and *Rs* (Figure 7), similar to observations that cell-free supernatants of *B. subtilis* and *B. amyloliquefaciens* inhibited the mycelial growth and/or spore germination of *Macrophomina phaseolina*, the causal agent of charcoal rot in soybean and common bean, and *Fusarium semitectum*, which causes rice dirty panicle disease, respectively [58,59,60]. Moreover, lipopeptide extract of *B. amyloliquefaciens* DHA55 at 100 µg/mL significantly inhibited *Fon* growth (Figure 7C), which was consistent with previous observations regarding *B. methyltrophicus* TEB1 and *B. amyloliquefaciens* SYBC H47 [49,61,62,63].

Various antifungal lipopeptides (e.g., iturin, iturinA, bacillomycin D, and fengycin) have been identified in *Bacillus* species with activities against different phytopathogenic fungi, such as *F. oxysporum*, *Fusarium verticillioides*, and *Botrytis cinerea* [48,64,65,66,67,68,69,70]. Similarly, *B. amyloliquefaciens* DHA55 produced four families of antifungal lipopeptides—iturin, bacillomycin D, surfactin, and fengycin (Figure 8)—which was further validated by detecting the genes responsible for the biosynthesis of the identified lipopeptides in *B. amyloliquefaciens* DHA55 (Table 3, Figure 8). The sequences of the lipopeptide biosynthesis genes in *B. amyloliquefaciens* DHA55 showed high levels of identity with those identified in other *Bacillus* species, such as *B. velezensis* and *B. amyloliquefaciens* [71,72,73]. Notably, the lipopeptide extract of *B. amyloliquefaciens* DHA55 exhibited broad-spectrum antifungal activity against three different pathogenic fungi (*Fon*, *Ss*, and *Rs*) with different lifestyles and infection processes (Figure 7), implying its significant potential to control various fungal diseases in different crops. Recently, it was found that myriocin from *B. amyloliquefaciens* LZN01 inhibited *Fon* by disrupting its cellular integrity [57]. However, the identification, purification, and characterization of the antifungal lipopeptides of *B. amyloliquefaciens* DHA55 that allow it to control plant diseases require further investigation.

Results from disease experiments showed that *B. amyloliquefaciens* DHA55 was the most effective strain in suppressing watermelon Fusarium wilt (Figure 5), possibly due to its capability to produce antifungal lipopeptides. This was consistent with previous observations of the different plant-protecting biochemical and physiological mechanisms of rhizobacteria against various pathogens [60,74,75]. It has been reported that rhizosphere *Bacillus* sp. strains colonize root and internal plant tissues [43,44], which was validated by morphological observations of the watermelon root–*Fon*–DHA55 strain system (Figure 9). Interestingly, SEM observations showed that *B. amyloliquefaciens* DHA55 adhered to and reproduced on the root surface (Figure 9). Importantly, *B. amyloliquefaciens* DHA55 interacted with and disintegrated the *Fon* mycelia on the root surface of watermelon plants (Figure 9G). This feature might be responsible for the direct inhibition of *Fon*, thus making it possible to suppress Fusarium wilt in watermelon, and correlates with previous observations reporting the destructive impact of *B. subtilis* and *B. amyloliquefaciens* on *F. oxysporum* and *Phytophthora capsici* on the root surface of cucumber [61,76]. Moreover, *B. subtilis* SQR 9 has been reported to colonize the root surface, which might be triggered by rhizodeposits exuded by host plants, competing with and suppressing the proliferation of fungal pathogens on the root surface of the host plants, such as soybean and mung bean [72,77]. Further investigation is needed to determine whether the rhizosphere chemistry affects the colonization of *B. amyloliquefaciens* DHA55 in the roots of watermelon plants. Alternatively, some rhizobacteria have been shown to suppress disease development by modulating plant defense responses, such as activation of induced systemic resistance [47,78]. For example, *B. amyloliquefaciens* PMB05, *B. subtilis* MBI600, and *B. cereus* EC9 have been reported to induce systemic resistance, contributing to the biocontrol of soil-borne pathogens, such as *F. oxysporum* [79,80,81,82]. Recently, it has been found that *B. velezensis* F21 enhances the basal immunity of watermelon plants against *Fon* by increasing the expression of the defense-related genes and activities of antioxidant enzymes, such as catalase, peroxidase, and superoxide dismutase [7]. Therefore, it is worth studying whether *B. amyloliquefaciens* DHA55 suppresses Fusarium wilt by priming the defense response in watermelon plants.

## 5. Conclusions

The present study established the antifungal activity of six antagonistic bacterial strains against five agronomically important phytopathogenic fungi; in particular, *Fon*. The results revealed that these strains have great potential as biocontrol agents for suppressing watermelon Fusarium wilt and promoting plant growth. Among these strains, *B. amyloliquefaciens* DHA55 exhibited the best performance in promoting plant growth and suppressing Fusarium wilt in watermelon. *B. amyloliquefaciens* DHA55 produced antifungal lipopeptides that showed significant inhibitory activity against *Fon* in vitro and on the root surface of watermelon plants. The present study highlights the potential of *B. amyloliquefaciens* DHA55 as a biopesticide for controlling Fusarium wilt in watermelon, offering an eco-friendly and cost-effective alternative to chemical fertilizers and pesticides. Overall, our study provides a basis for the further development of biocontrol strategies based on *B. amyloliquefaciens* DHA55 to combat watermelon Fusarium wilt in the field.

## Figures and Tables

**Table 1:** Primers for PCR detection of the lipopeptide biosynthesis genes in *Bacillus amyloliquefaciens* DHA55.

Lipopeptides	Primers	Sequence (5′–3′)	Genes	Size (bp)
Iturin	BamB1F	CGACATACAGTTCTCCCCGGT	*ItuB*	473
	BamB1R	AAGAAGGCGTTTTTCAAGCA		
	ITUCF1	TACGGAGGAGAAAACAGTGC	*ItuC*	450
	ITUCR3	ACCTCTGGCACAAAGGGGTG		
	ItuD2F	GACGGTAGATTCGCTGCTGT	*ItuD*	593
	ItuD2R	TGATGCGATCTCCTTGGATG		
Fengycin	FNDF1	CTGGGAGGTCAGCCGGTCTG	*FenD*	167
	FNDR1	GTGGTCGCCGGTTCACAAAT		
	FenB1F	CCCTTGTCAGAAACAGCAAT	*FenE*	704
	FenB1R	GCTTCTATTTCGGCAGGCTC		
Surfactin	110F	TATAAGCCGGCAGCGAGCTG	*SrfAB*	202
	110R	GAGTGTCTGTTTCCAAATGC		

**Table 2:** Characterization of the screened antagonistic bacterial strains.

Strains	Shape	Gram Staining	Cat. ^a^	Prot. ^a^	Cel. ^a^	Amm. ^a^	HCN ^a^	PSI (mm) ^a,b^	Siderophore (mm) ^b^	IAA (μg/mL) ^a^
DHA4	Rod	+	+	+	+	+	−	0.38 ± 0.01	0.53 ± 0.19	394.90
DHA6	Rod	+	+	+	+	+	−	0.45 ± 0.07	0.51 ± 0.19	420.05
DHA10	Rod	+	+	+	+	+	−	0.36 ± 0.01	0.49 ± 0.19	327.19
DHA12	Rod	+	+	+	+	+	−	0.36 ± 0.01	0.49 ± 0.18	165.05
DHA41	Rod	+	+	+	+	+	−	0.38 ± 0.01	0.49 ± 0.18	311.36
DHA55	Rod	+	+	+	+	+	−	0.40 ± 0.01	0.54 ± 0.20	393.14

^a^ Cat., catalase; Prot., protease; Cel., cellulase; Amm., ammonium; HCN, hydrogen cyanide; PSI, phosphate solubilizing index; IAA, indole-3-acetic acid. ^b^ means ± standard deviation from three independent experiments. +, Positive; −, negative.

**Table 3:** Sequence similarities of the specific lipopeptide biosynthesis genes in *Bacillus amyloliquefaciens* DHA55.

Genes	Size (bp)	Best Matches in GenBank	Organism/Isolate for Best Matches	BlastN *e* Value	Nucleotide Identity (%)
*Iturin C*	450	ALA39967.1	*B. amyloliquefaciens*	0.0	98.60
*Iturin B*	473	MBM7358087.1	*B. velezensis*	0.0	98.67
*Iturin D*	493	QBY06353.1	*B. amyloliquefaciens*	0.0	95.81
*Fengycin E*	704	ACX55806.1	*B. amyloliquefaciens*	0.0	97.31
*Fengycin D*	167	AGU42446.1	*B. amyloliquefaciens*	8 × 10^−79^	100
*Surfactin*	202	QPC9684.1	*B. velezensis*	3 × 10^−89^	92.31

**Table 4:** Cellular fatty acid content in *Bacillus amyloliquefaciens* DHA55.

C14:0 iso	C15:0 iso	C15:0 anteiso	C16:0 iso	C16:0	C17:0 iso	C17:0 anteiso
1.65	15.20	32.76	15.20	13.27	5.93	6.79

## Data Availability

All the data are present in the manuscript.

## Supplementary Materials

The following supporting information can be downloaded at: https://www.mdpi.com/article/10.3390/jof9030336/s1, Figure S1: Detection of the lipopeptide biosynthesis genes from *Bacillus amyloliquefaciens* DHA55.
