# Supplementary material for: Suppression of Fusarium Wilt in Watermelon by Bacillus amyloliquefaciens DHA55 through Extracellular Production of Antifungal Lipopeptides
Source: J Fungi (Basel). 2023 Mar 9;9(3):336. doi: 10.3390/jof9030336 (PMC10053319; doi:10.3390/jof9030336)
Supplement: Supplementary file 1 [file jof-09-00336-s001.zip › jof-2255318-supplementary.pdf]

Supplementary Material

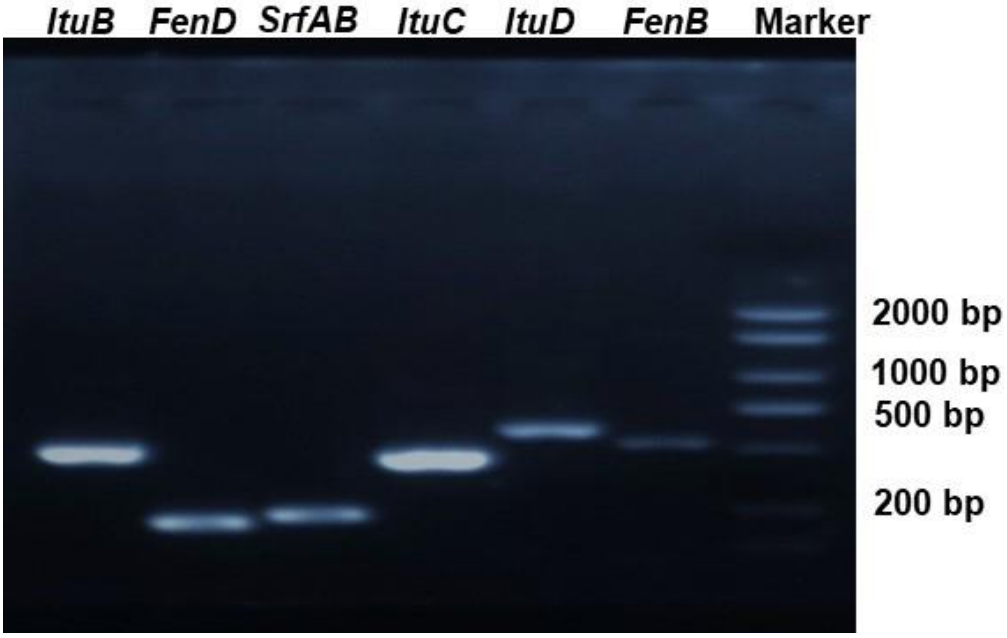

Figure S1. Detection of the lipopeptide biosynthesis genes from *Bacillus amyloliquefaciens* DHA55. Lane 1, ItuB; Lane 2, FenD; Lane 3, SrfAB; Lane 4, ituC; Lane 5, ituD; Lane 6, FenB; and DL, 2000 DNA marker.
